# 

**Published:** 1876-07

**Authors:** 


### Books and Pamphlets Received.

---

Atlas of Skin Diseases. By Louis A. Duhring, M. D., etc. Part I. Eczema, Psoriasis, Lupus Erythematosus, and Syphiolderma. Philadelphia: J. B. Lippincott & Co., 1876. Buffalo: Theo. Butler & Son.

A Practical Treatise on Materia Medica and Therapeutics. By Robert Bartholow, M. A., M. D. New York: D. Appleton & Co., 1876 Buffalo: Herger & Ulbrich.

Inhalation in the Treatment of Disease, its Therapeutics and Practice. A Treatise on the Inhalation of Gases, Vapors, Fumes Compressed and Rarified Air, Nebulized Fluids and Powders. By J. Solis Cohen, M. D. Philadelphia: Lindsay & Blakiston, 1876. Buffalo: Theo. Butler & Son.

The Students Guide to Dental Anatomy and Surgery. By Henry Sewill. Philadelphia: Lindsay & Blakiston, 1876. Buffalo: Theo. Butler & Son.

The Student's Guide to the Practice of Midwifery. By Lloyd D. Roberts, M. D. Philadelphia: Lindsay & Blakiston, 1876. Buffalo: Theo. Butler & Son.

Micro-Photographs in Histology, Normal and Pathological, No. 3. By Carl Seiler, M. D., in conjunction with J. G. Hunt, M. D., and J. G. Richardson, M. D. Philadelphia: J. H. Coats & Co.

Specimen Fasciculus of the Catalogue of the National Medical Library under the Direction of the Surgeon General, United States Army, at Washington, D. C.

---

Messrs. Hurd & Houghton, announces a work on the Anatomy of the Head. By Thomas Dwight, M. D., they also announce the second volume of Public Health, containing the most important papers read at the American Public Health Association.

### INDEX.

### A

Abbott, F. W., M. D. The Cornea in Health and Disease, ... 41
Abstract of the Proceedings of the Buffalo Medical Association, ... 12, 49, 191, 225, 343, 385, 420, 466
Address, Inaugural, of the President of the Medical Society of the State of New York, Dr. Thomas F. Rochester, delivered in Albany, June 20th, 1876, ... 401
Address, Introductory. Delivered at the Buffalo Medical College Nov. 3, 1875, by Prof. E V. Stoddard, M. D., ... 121
Albany County Medical Society, Report of Semi-Monthly Meetings ... 136, 215, 267, 371
American Medical Association, Twenty-Seventh Annual Meeting of, ... 429
Aphonia, Notes of a Case of, Treated by the Method of Oliver. By W. W. Potter, M. D. ... 135
Apoplexy, Artificial Respiration in, ... 25
Articular Rheumatism, Alteration of Susceptibility in, and Electro-Therapeutic Treatment, ... 312

### B

Blackham, Geo. E., M. D. Notes on Microscopy, ... 66
Bladder. Extirpation of a Tumor of, ... 23
Bloodless Operations, Duration of, ... 113
Books and Pamphlets Received, ... 40, 80, 120, 160, 240, 280, 320, 360, 400, 440, 474
Brush, E. N., M. D. Notes on Obstetrics and Gynæcology, ... 70
Brush, E. N., M. D. Notes on the Treatment of Syphilis, ... 19
Brush, E. N., M. D. On Unreduced Dislocations of the Shoulder, with Notes of two Cases, one of Two Hundred and thirty days, and one Congenital, of eleven months, reduced by Prof. J. F. Miner, M. D. ... 81
Brush, E. N., M. D. Two Cases of Removal of Omental Tumors from the Scrotum. By Prof. J. F. Miner, M. D. Report of, ... 9
Buffalo Medical Association, Abstract of the Proceedings of, ... 12, 49, 191, 225, 343. 385, 420, 466
Buckley, L. Duncan, M. D. Clinical Conversations on Diseases of the Skin, ... 321

### C

Camphor Poisoning, ... 114
Carotid Artery, Ligation for Injury of. Report of Case By E. T. Dorland, M. D. 298
Chloral Hydrate, The Local use of ... 29
Chloralism, ... 111
Chloral, Poisoning by, ... 33, 234
Chorea, By Henry Nichell, M. D. ... 100
Clarke, O. H. E., M. D. The Surgical Treatment of Dysmenorrhœa, ... 441
Cornea in Health and Disease, By F. W. Abbott, M. D. ... 41
Croup and Diphtheria, The non-identity of as shown by their Pathological and Histological Anatomy, ... 392
Curtis, F. C., M. D. Certain Points connected with Typhoid Fever, ... 255
Cystitis, Treatment of Chronic, ... 109

### D

Diehl, Conrad, M. D. Ex-section of the Femur for Gun-shot injury, ... 342
Dimon, Theodore, M. D. The Influence of Medical Studies upon Religious Belief, ... 54
Diseases of the Skin, Clinical Conversations on, By L. Duncan Buckley M. D. 321
Dorland, E. T., M. D. Ligation of the Carotid Artery for Injury ... 298
Dysmenorrhœa, The Surgical Treatment of, By O. H. E. Clarke, M. D. ... 441

### E

EDITORIAL.
- Alumni Association of the Medical Department of the University of Buffalo, ... 237, 274
- American Dermatological Association, 471
- American Medical Association, ... 315, 435
- An Unmitigated Quack, ... 77
- Close of Volume Fifteen, ... 470
- Foreign Quacks and American Medical Colleges, ... 353
- Homœopathy in the Michigan University, ... 73, 355
- In Memoriam, ... 317
- International Medical Congress, ... 235, 313, 469
- Meeting of Medical Societies, ... 399
- Meeting of the American Pharmaceutical Association, ... 78
- Meeting of the New York State Medical Society, ... 438
- Meeting of the Representatives of American Medical Colleges ... 437
- New Years, ... 200
- Notes, ... 78, 238, 315
- Notice, ... 34
- Physicians and Life Insurance Companies, ... 119
- Preliminary Education of Medical Students, ... 396
- Sanitary Condition of Philadelphia, ... 397
- Volume Fifteen, ... 34

Effusions. Pleuritic, The Operative Treatment of, By Herman Mynter, M. D. ... 377
Enteric Fever and Milk Supply ... 107
Etiology of Elephanteasis, Endemic Hæmaturia and Chyluria, By Charles H. Richmond, M. D. ... 281

(477)

Excision of the Knee for Chronic Disease of the Joint, ---- ---- ------------ ---- 27
Exercise as a Remedial Agent, By H. R. Hopkins, M. D. ----------- ----------- 289
Exudations, Pleuritic, The Operative Treatment of, ---------------------------- 889

**F**

Family Physicians and Life Insurance Companies, --- --------------------------- 115
Femur, Ex-section of, for Gun-shot Injury, By Conrad Diehl. M D --------------- 342
Forceps, Against the Pendulum movement in working the, ---- ------------------ 304
Fracture, Plaster of Paris treatment of, -- 104

**G**

Gay, C. C. F., M. D. Notes of Surgical Cases, ------------------------------ 301, 416
George, Conrad, M. D. Physiological Considerations in Transfusion of Blood, ---- 361

**H**

High Temperature, Mr. Teale's Case, ----- 113
Hopkins. H. R., M. D Exercises as a Remedial Agent, ------------------------ 289
Howe, Lucien M. D. Notes on Ophthalmology, -------------------------------- 144
Howe, Lucien. M. D. The Influence of Various Occupations upon the Eye, ---- 1
Hyde. Frederick E., M. D. On the Use Warm and Hot Water in Surgery, ------ 161, 201, 241

**M**

Medical Colleges, Proceedings of the Association of Representatives of, -------- 424
Medical Studies, Their Influence upon Religious Belief. By Theodore Dimon, M. D., ---------- -- ---------- 54
Medical Study, Neglected Branches of ---- 154
Miner. W. W., M. D. Chronic Dislocation of the Shoulder, ------ ----- -- 181
Miner, W. W., M. D. Notes on Surgery, - 15
Mynter, Herman, M D. The Operative treatment of Pleuritic Effusions, ------- 377

**N**

Nichell, Henry, M. D. On Chorea, ------- 100
Notes of Surgical Cases, By C. C. F. Gay, M. D., ----------------------------- 301, 416
Notes on Microscopy. By Geo. E. Blackham, M. D. -------------------------- 66
Notes on Obstetrics and Gynæcology. By E. N. Brush, M. D. -------------- 70
Notes on Ophthalmology. By Lucien Howe, M. D. ------------------------ 144
Notes on Surgery, By W. W. Miner, M. D. 15
Notes on the Treatment of Syphilis, By E. N. Brush, M. D. -------------------- 19

**O**

Obituary.—Dr. M. E. Potter, ------------- 156
Occlusion of the Vagina Consequent upon an Abortion, ---- -------------------- -- 152
Omental Tumor, Two Cases of Removal of, from the Scrotum, By J F. Miner, M. D. Reported by E N. Brush, M. D. 9
Ovariotomy, Menstruation from the Pedical, ----------------------------------- 350

**P**

Paraplegia, Diagnosis of the Lesion in ---- 146
Potter, W. W., M. D. Hemorrhage following Miscarriage, and its treatment by Intra-Uterine Styptic injections, -------- 414
Potter, W. W., M. D. Notes on a Case of Aphonia, Treated by the Method of Oliver, ---------------------------------------- 135
Puerperal Fever, Epidemic, ------------- 229

**R**

Reviews.
A Manual of Diet in Health and Disease. By Thomas King Chambers, M. D, F.R.C.P., --------------- 79
A Manual of Pathology. By Ernst Wagner, ------------- ----------- 439
A Report on the Hygiene of the U. S. Army, with a Description of the Military Posts, ----------------- 79
A System of Midwifery. By William Leishman, M. D. ---- ------- 319
A Text-Book of Human Physiology. By Austin Flint, Jr., M. D. -------- 279
A Treatise on Human Physiology. By John C. Dalton, M. D ------ ---- 158
A Treatise on the Diseases of Infancy and Childhood. By J Lewis Smith, M. D. ------------------------------ 357
A Treatise on the Diseases of the Nervous System. By Wm. H. Hammond, M. D. ------------------------------ 474
Clinical Lectures and Essays. By Sir James Paget, Bart, -------------- 239
Clinical Lectures on Diseases of the Urinary Organs. By Sir Henry Thompson, ------------------ -- 36
Cyclopædia of the Practice of Medicine. By Dr. Hoon Ziemssen, -- -- 35, 117, 277
Extra-Uterine Pregnancy. By John S. Parry, M. D. -------------- -- 358
Filth Diseases and their Prevention. By John Simon, M D. ------------ 472
Hospital Plans, A Series of Five Essays Relating to the Construction, etc., of Hospitals, ------------------ 400
Lectures on Diseases of the Nervous System, By Jerome K. Baudny, M. D. ------------------------------- 239
Lectures on Syphilis. By Henry Lee, 278
Lindsay and Blakiston's Visiting List for 1876, ---------------------- . 160
On Functional Derangements of the Liver, By Charles E. Murchison, M. D., F. L. D., F.R.S. ---------- 37
On Paralysis from Brain Disease in its Common Forms. By H. Charlton Bastian, M. A., M. D, F.R.S. ---- - 158
On Poisons in Relation to Medical Jurisprudence and Medicine. By Alfred Swaine Taylor. M. D, F.R.S. 279
Phthisis. By Austin Flint, M. D. ---- 318
Sex in Industry. By Azel Ames, M. D. 38
The Archives of Dermatology -------- 357
The Medical Jurisprudence of Insanity. By J. H. Balfour Browne, Esq. 317
The Mucous Membrane of the Uterus. By Geo. J. Engelman. A. M., M. D. 159
Transactions of the College of Physicians of Philadelphia, --- --------- 157
Vision, Its Optical Defects and the Adaption of Spectacles. By C. S. Fenner, M. D. ---- - ------- 119
Richmond, Charles H., M. D. The Etiology of Elephanteasis Endemic Hæmaturia and Chyluria, -------------------- 281

(478)

Rochester, Thomas F., M. D. Inaugural address as President of the Medical Society of the State of New York; Delivered in Albany, June 20th, 1876. 400

**S**

Shoulder, Chronic Dislocation of the, By W. W. Miner, M. D. 181
Shoulder-Joint. On Unreduced Dislocations of the, With report of two Cases, One of Two Hundred and thirty days, and One Congenital, of eleven months. Reduced by Prof. J. F. Miner, M. D., with Remarks. By E. N. Brush, M. D. 81
Stoddard, Prof. E. V., M. D. Introductory Address delivered at the Buffalo Medical College, Nov. 3, 1875, 121
Strangulation of the Intestine by Cicatrices, 228
Sympathetic, Nervous System. By S. W. Wetmore, M. D. 328
Sympathetic Ophthalmia, 30
Syphilis, The Influence of, In Pregnant Women, 32
Syphilitic Patients, Effects produced upon the Blood by Treatment with Mercury, 308

**T**

The Influence of Various Occupations upon the Eye. By Lucien Howe, M. D. 1
Tracheotomy, 272
Transfusion of Blood, Physiological Considerations in, By Conrad George, M. D. 361
Typhoid Fever, Certain Points connected with, By F. C. Curtis, M. D. 255

**U**

Unsuccessful Practitioner, The Experiences of 59, 102
Urinary Retention 66
Uterine Injection, Sudden death after, 227
Uterine Injection in Hemorrhage following Miscarriage, By W. W. Potter, M. D. 414
Uterus, Fibroids of the, On the Actual Cautery in the Enucleation of 273
Uterus Inversion of, with operation by White's Method, 232

**W**

Water. Warm and Hot in Surgery, By F. E. Hyde, M. D. 161, 201, 241
Water, Use of, in Typhoid Fever, 28
Wetmore, S. W., M. D. The Sympathetic Nervous System, 328

